# Use of proton pump inhibitors and increased risk of fracture in type 2 DM and menopausal women: a systematic review and meta-analysis

**DOI:** 10.4314/ahs.v26i1.15

**Published:** 2026-03

**Authors:** Pradeep Battula, Poli Bhuvana Sree, Yapareddygari Sandhya Latha, Kotha Naga Poojitha, Kamini Venkata Subba Reddy, Setlem Sai Venkata Krishna, P Raghu Sri Charan, Krishna Undela

**Affiliations:** 1 Department of Pharmacy Practice, Santhiram College of Pharmacy, Nandyal, Andhra Pradesh, India; 2 Department of Surgical Gastroenterology, Santhiram medical college and General Hospital, Nandyal, Andhra Pradesh, India; 3 Department of Pharmacy Practice, National Institute of Pharmaceutical Education and Research (NIPER) Guwahati, Assam, India

**Keywords:** Elderly patients, Proton pump inhibitors, Postmenopausal women, Type 2 diabetes mellitus, Risk of fractures

## Abstract

**Objective:**

To investigate the relationship between type 2 diabetes mellitus and postmenopausal women with fracture risk when using PPIs.

**Methods:**

We conducted an observational study to investigate the association between PPI use and an increased fracture risk in postmenopausal women and type 2 diabetes mellitus. We searched for epidemiological studies published between 2006 and 2023 in PubMed, Google Scholar, and other bibliographies of the retrieved papers. The pooled OR was obtained using the random-effects model, and a subgroup analysis was conducted.

**Results:**

Eight studies, including six cohorts and two case-control studies, were conducted with 1.3 million participants. In these studies, 1,63,346 cases of fracture risk were identified. The pooled effect estimate of these studies was OR 1.61, with a 95% CI of 1.36-1.90. This indicates a significant correlation between Type 2 diabetes mellitus, post-menopausal women, and the risk of fractures. The OR for type 2 DM alone was 1.34 (95% CI 1.14-1.57), and for menopausal women alone, the OR was 2.15 (95% CI 1.36-1.90). Notably, there was no evidence of publication bias.

**Conclusion:**

This meta-analysis provides strong evidence that PPI use in type 2 DM and menopausal women is significantly associated with the risk of developing fractures.

## Introduction

Globally, proton pump inhibitors (PPIs) are widely prescribed for the treatment of peptic ulcers, reflux, and heartburn by inhibiting gastric acid production more effectively than H2 blockers^1^. PPIs block the gastric acid secretion pump in parietal cells, deactivating it irreversibly, as the acid environment converts the drug from an inactive form to an active form and binds covalently to the pump^2^.

Proton pumps are crucial for stomach acidity and are effective in alleviating indigestion and heartburn. However, its inhibition can cause hypochlorhydria, hypomagnesemia, and vitamin B12 malabsorption due to reduced acid secretion, affecting nutrient absorption^3^. The current evidence suggests that PPIs are often overused, with 25-70% of prescriptions having no appropriate indications^4^. Increased usages of proton pump inhibitors increase the risk of fractures, particularly in older individuals with conditions such as Rheumatoid Arthritis, Osteoporosis, Type 2 DM, and post-menopausal women. This heightened risk stems from reduced acidity, hindering calcium absorption, and exacerbating fracture vulnerability^5^. Most fractures in persons 50 or older are caused by osteoporosis or osteopenia^5^. PPIs and fractures have been repeatedly linked in persons who have osteoporosis or are at high risk for developing osteoporosis^6^.

Approximately 200 million patients worldwide suffer from osteoporotic hip fractures linked to various risk factors including glucocorticoids, lower bone quality, and aging. Falls or everyday tasks can lead to fractures when the weaker bones are overloaded^7^.

Numerous observational studies have linked proton pump inhibitors to an increased risk of fracture. In 2010, the US Food and Drug Administration issued an alert warning of increased fracture risk in patients taking high-dose or long-term PPIs^8^.

As life expectancy increases, women spend one-third of their lives in menopause. Half of postmenopausal women endure osteoporosis-related fractures, leading to increased mortality risk, reduced quality of life, persistent pain, disability, and loss of independence^9^-^10^.

Individuals with type 2 diabetes mellitus have a greater risk of fracture owing to structural bone abnormalities associated with vascular and neurological disorders.

Furthermore, Proton Pump Inhibitors (PPIs) reduce bone strength by inducing calcium malabsorption, hypomagnesemia, and hypochlorhydria, which increases gastric pH and makes bones brittle^11^.

Diabetes mellitus frequently affects various bodily systems, including muscles, retina in the eyes, endocrine system, particularly parathyroid hormone (PTH) and estrogenic cytokines, nervous system, and digestive system. Diabetes lowers bone synthesis and accelerates bone resorption by negatively regulating osteoblast differentiation and function and positively regulating osteoclast differentiation and function via various intermediary factors^12^.

Meta-analysis is a statistical method used in social science research to combine and analyze the results of multiple studies. Several observational and prospective studies have investigated the relationship between PPI use and fracture risk. A large number of incident cases may increase the statistical power of meta-analysis in determining the association between exposure and outcome risk^13^-^14^.

Given the conflicting published literature and heterogeneity of research studies, we conducted a systematic literature review and meta-analysis to further investigate the relationship between PPI use and the risk of hip fracture.

## Methodology

The present study followed the Cochrane Collaboration guidelines for conducting systematic reviews and meta-analyses, and the Preferred Reporting Items for Systematic Reviews and Meta-analysis (PRISMA) statement was used as a guideline for preparing the present study.

### Literature Search

A comprehensive literature search was performed using electronic databases, including PUBMED, GOOGLE SCHOLAR, and PROQUEST, for observational cohort and case-control studies investigating the potential link between the long-term use of PPIs for menopausal women and type 2 DM and the risk of fractures. Each database was searched using the following terms: type 2 DM, menopausal women, older women, long-term PPIs, fractures, incidence, and bone density. We searched for additional studies in the bibliographies and citation sections of retrieved articles. The reference lists of the potential articles were manually searched for additional eligible articles.

### Study Selection

Two members independently reviewed the titles and abstracts of all the identified citations according to the inclusion criteria. After reading the titles and abstracts, the full texts were retrieved. Any discrepancies were rectified through communication with the third author. An abstract from an epidemiological study (cohort or case-control) that reported the original data and was published in English was considered relevant. The outcome variable was fracture risk and the predictor variable was long-term PPI use in conditions related to the disease (menopausal women, type 2 DM).

Peer-reviewed studies were included if they met PICO criteria.

Types of studies: observational studies (cohort, case-control)
P (types of participants): type 2 DM and older women experiencing menopause with any fracture.
I (Type of intervention): The experimental intervention included long-term PPI use before fracture development.
C (Type of comparison): Comparison between PPI users and non-users in type 2 DM and menopausal women.
O (type of outcome): To estimate risk, a list of fracture outcomes reported in the studies, including the odds ratios (ORs), 95% confidence intervals (CIs), or other appropriate data, was compiled.

### Data Extraction and Quality Assessment

A prespecified data extraction template was used to gather information from each qualified study. The following information was extracted from each eligible study: study design, study period, publication year, study author and country, ascertainment of PPI exposure, fracture assessment, study population, statistical adjustments, and study quality. Each study chosen was subjected to independent data abstraction by two reviewers, and any dispute between them was resolved by a conversation with a senior investigator. Two independent reviewers performed quality assessments. The quality of the included observational studies was assessed using a modified version of the Newcastle–Ottawa Scale (NOS) recommended by Cochrane. The NOS system follows a star-based evaluation method (up to nine stars) to assess the quality of a study in three key areas: participant selection, comparability of study groups, and determination of the desired outcomes. We can consider the study quality based on the star system as low-risk and high-risk, if it receives a score of greater than or equal to 7 (high quality) and <7 (low quality). This information is presented in Tables 1 and 2, respectively.

**Table 1:** The Methodological quality of case-control studies based on the Newcastle-Ottawa scale (N=2)

	Selection			Comparability	Exposure				
**Study**	**Adequate definition of cases**	**Representativeness of cases**	**Selection of controls**	**Definition of controls**	**Comparability of cases and controls based on design or analysis**	**Ascertainment of exposure**	**Same method of ascertainment for cases and controls**	**Non-response rate**	**Total score**
**Douglas et al., (2010)**	1	1	1	0	2	1	2	1	9
**Hui Fen Chiu et al., (2010)**	1	1	1	1	2	1	1	1	9

**Table 2:** The methodological quality of cohort studies based on the Newcastle-Ottawa scale(N=6)

Selection				Comparability	Outcome				
**Study**	**Representativenss of the exposed cohort**	**Selection of the non-exposed cohort**	**Ascertainment of exposure**	**Demonstration that the outcome of interest was not present at the start of the study**	**Comparability of the cohort based on the design or analysis**	**Assessment of outcome**	**Was the follow-up long enough for an outcome to occur**	**Adequacy of follow-up of cohorts**	**Total score**
**Ya-shuan Chou et al., (2020)**	1	1	1	1	1	1	1	0	7
**Lewis et al., (2014)**	1	1	1	1	1	1	1	0	7
**Khalili et al., (2012)**	1	1	1	1	1	1	1	0	7
**Shelly L.gray et al., (2010)**	1	1	1	1	2	1	1	0	8
**Targowink et al., (2008)**	1	1	1	1	1	1	1	1	8
**Roux et al., (2008)**	1	1	1	1	2	1	1	0	8

### Statistical Analysis

The main aim of the study was to determine the combined odds ratio, along with a 95% confidence interval, for the likelihood of fractures in menopausal women with type 2 diabetes who took PPIs compared to those who did not use PPIs. The summary odds ratio (OR) with 95% CI from HRs, ORs, and RR was calculated to measure the magnitude of fractures with PPI use in associated disease conditions (type 2 DM and menopausal women). The outcome of interest was rare; therefore, we considered the OR equivalent to RR. Effect estimates were pooled using the Der-Simonian and Laird random-effects models, accounting for both within-study and between-study variances. The overall OR with 95% confidence intervals (CIs) was obtained using generic inverse variance weighting. We calculated the odds ratio using raw data. An odds ratio >1 indicates an increased risk of fracture, and <1 indicates a decreased risk of fracture^24^.

We assessed the statistical significance using an overall effect estimate (OR). A forest plot was drawn to evaluate Higgins's I^2^ value and quantify the heterogeneity among studies, including overall and within-group heterogeneity. An I^2^ value of 0–25% represents very low heterogeneity, 25-50% represents low heterogeneity, 50-75% represents medium heterogeneity, and > 75% represents high heterogeneity^24^. Cochrane's Q statistics, p<0.10 were considered statistically significant for heterogeneity.

Publication bias was assessed by examining the funnel plot and further confirmed using Egger's regression test and Harbord's and Peters' regression tests. If a p<0.05, it is considered statistically significant. Stratified analyses were conducted to determine whether differences in study design (cohort, case-control), ethnicity (Asian, Western), fracture site (hip, vertebral), and quality assessment identified essential subgroups. All statistical analyses were conducted using version 29.0.0.2.0 [20] of the IBM SPSS (Statistical Package for the Social Sciences).

### Search Results

**Figure 1:**
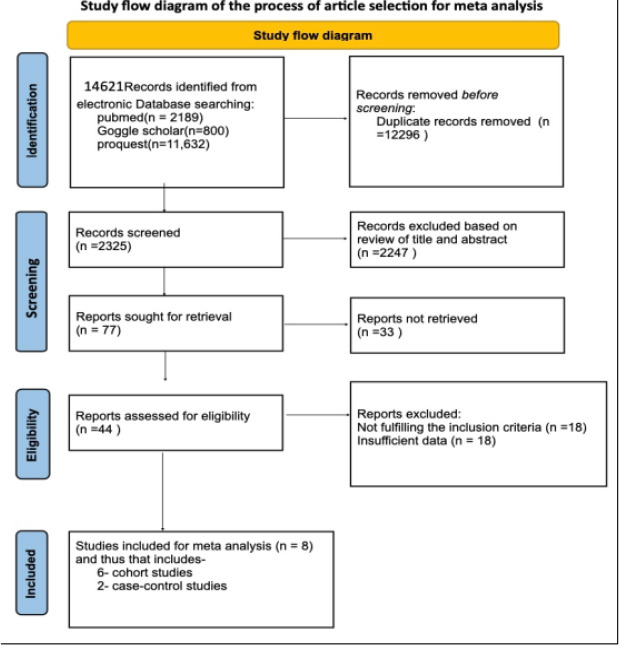
Flow chart of the studies considered and finally selected for review

Three electronic databases yielded 14621 records. After removing 12296 duplicate records and excluding 2247 records based on a review of their titles and abstracts, only 77 records remained. Finally, eight studies were included in the meta-analysis after excluding data that did not meet the inclusion criteria or had insufficient data. The number of articles excluded at each stage of the eligibility assessment is shown in Figure 1.

### Characteristics of the Included Studies

Table 3 presents the characteristics of the studies included in this analysis. of the eight studies, six were cohort studies, and two were case-control studies. The sample size of each study ranged from 1045 to 567586, with a pooled sample size of 1359115. Four of the eight studies evaluated the effect of PPI on postmenopausal women, while the remaining four assessed impact of PPI on DM separately.

**Table 3:** The study characteristics were included in the final analysis (N=8)

Study author (Year)	Country	Study design	Study population	Age (years)	Cases (n)	Controls (n)	Assessment of exposure (PPIs)	Assessment of fracture	Effect estimates (OR, HR, RR)	Model used	Adjustments	NOS scale
**Roux et al, (2009)**	Germany and France	prospective cohort	1211 subects in post-menopausal women	65.9yrs	61	1150	Any PPI use and non-PPI use	Hip fractures	Overall Adjusted HR-1.41(95%CI) 1.29-1.54	Random-effect model	Adjusted for age, history of fracture and comorbidities	8
**Shelly L. Gray et al, (2010)**	North America	Nested case control	161806 subjects of post-menopausal women	50-79yrs	3396	158410	PPI prescription based on the WHI database	Vertebral fractures	Overall Adjusted HR-1.00 (95% CI) 0.71-1.40	Random-effects mode	Adjusted for age, parental history of fractures and comorbidities	8
**Hameed Khalili et al., (2012)**	United states	Prospective cohort	565786 post-menopausal women	42.5(30-55)	79899	485887	Any PPI use and non-PPI use	Hip fractures	Overall Adjusted HR-1.35 (95% CI) 1.31-1.62	Random-effects mode	Adjusted for age, gender, comorbidities, and site of fractures	7
**Ya-Shuan Chou et al., (2020)**	Taiwan	Retrospective cohort	399481 subjects of type 2 DM	65 yrs	44341	177364	Newly diagnosed T2DM patients with PPI use >18yrs (PPI dose <28DED)	Hip fractures	Overall Adjusted HR-1.41 (95% CI) 1.29 - 1.54	Random-effects mode	Adjusted for age, gender, and comorbidities	7
**Lewis et al., (2014)**	Australia	Prospective cohort	1045 Subjects	80-82 yrs	120	925	Any PPI use and non-PPI use	Hip fractures	Overall Adjusted OR-2.17 (95%CI) 1.25-3.77	Random-effects mode	Adjusted for age, ethnicity, and other site of fractures	7
**Douglas et al., (2010)**	United states	Case control	164,223 post-menopausal women	>50 yrs	33752	130471	KPNC prescription pharmacy databases	Hip fractures	Overall Adjusted OR-1.30 (95% CI) 1.21-1.39	Random-effects mode	Adjusted for ethnicity, comorbidities, and medications are not included in the final model.	9
**Hui-Fen Chiu et al., (2010)**	Taiwan	Case control	Taiwanese women (2482)	75 yrs	1241	1241	NHRI prescription database with PPI usage	Vertebral fractures	Overall Adjusted OR-1.04 (95% CI) 0.73 - 1.49	Random-effects mode	Adjusted for age, gender, medications, index date, and comorbidities not included in the final model	9
**Targownik et al., (2008)**	Canada	Case control	Canadian women (63,081)	70-80 yrs	5361	16051	Drug program Information network database	Hip fractures	Overall Adjusted OR-1.92 (95% CI) 1.16-3.18	Random-effects model	Adjusted for age, gender, ethnicity, comorbidities, region and medications	8

As shown in Table 3, eight research studies were involved: six were conducted in Western countries and two in Asian countries. The quality of each study was assessed using the NOS scale, which ranges from six to nine points. This finding suggests that the observational studies were of reasonably good quality. Thus, all eight studies were of high quality (>7); however, we divided the score into high-high quality if the score was 9, high-medium quality if the score was 8, and high-low quality if the score was 7. The breakdown of the scores is provided in Tables 1 and 2.

## Results

### Primary Outcomes

**Figure 2:**
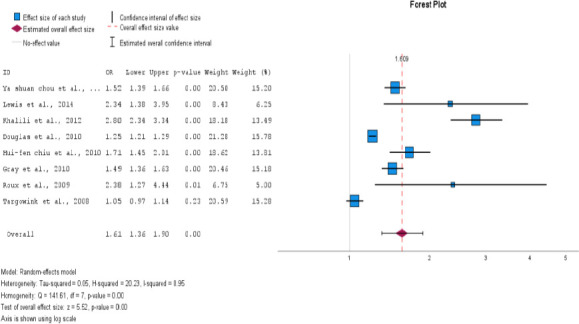
Use of proton pump inhibitors and increased risk of fracture in type 2 DM and menopausal women. The pooled estimate of the odds ratio [OR] and 95% confidence interval [CIs] of the total use of PPI in type 2 DM and menopausal women increased the risk of fracture. Based on eight studies (six cohorts and two case-control studies). The square size was proportional to the weight of the corresponding study in the meta-analysis; the length of the horizontal lines represents the 95% CI. The unshaped diamond indicates the pooled OR and 95% CI [random-effects model]

**Figure 3:**
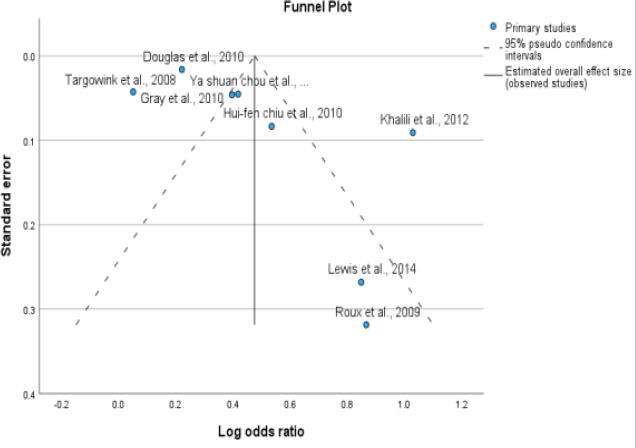
Assessment of publication bias. Funnel plot (publication bias assessment plot) of the log odds ratio of the risk of developing fractures by the standard error for all studies. Circles: studies included in the meta-analysis; ORs are displayed on a logarithmic scale. p = 0.45 for Egger's test

Our meta-analysis of eight studies showed a significant association between PPI exposure in type 2 DM and post-menopausal women and an increase in fractures. The overall odds ratio was 1.61 (95% CI=1.36- 1.90), with a p<0.001. Although high heterogeneity was observed among the studies (I2 = 95.1%), using a random effects model instead of a fixed effects model for the pooled estimate calculation provided us with a more accurate representation of the findings. These results highlight the importance of caution when using PPIs in type 2 DM and postmenopausal women, as they may increase the risk of fractures. This is displayed as a Forest plot in Figure 2 and a funnel plot for publication bias in Figure 3 for all studies.

### Subgroup Analysis

**Figure 4:**
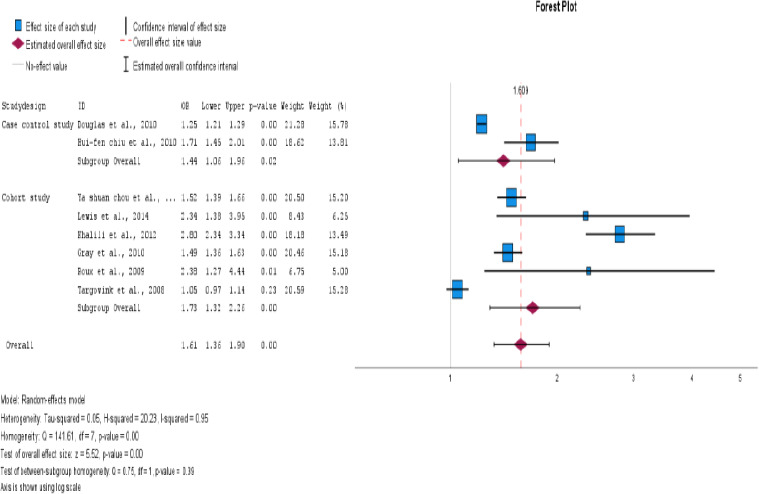
Subgroup analysis based on study design

**Figure 5:**
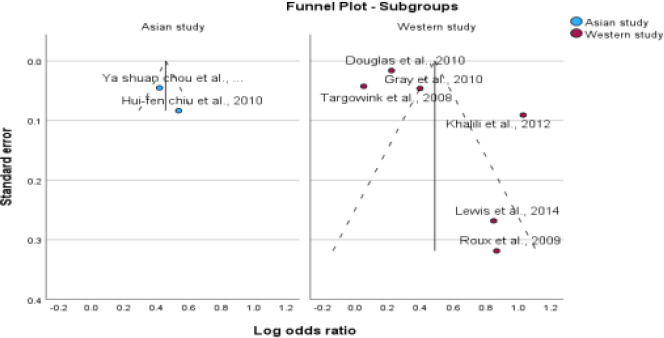
Subgroup analysis based on study design

**Figure 6:**
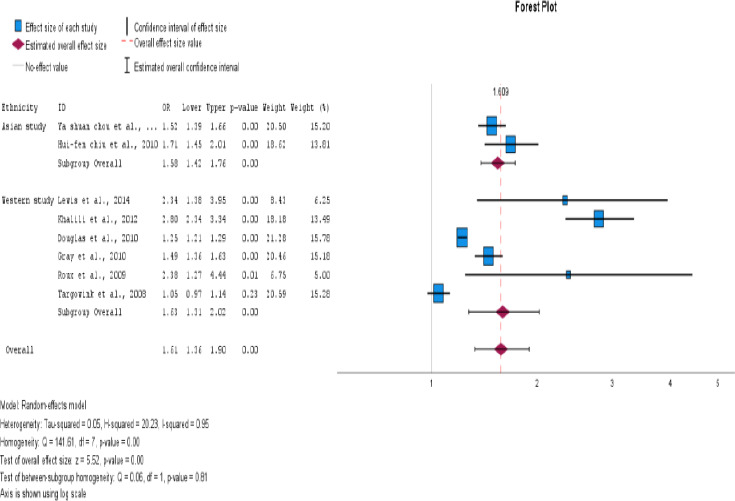
Subgroup analysis based on ethnicity

**Figure 7:**
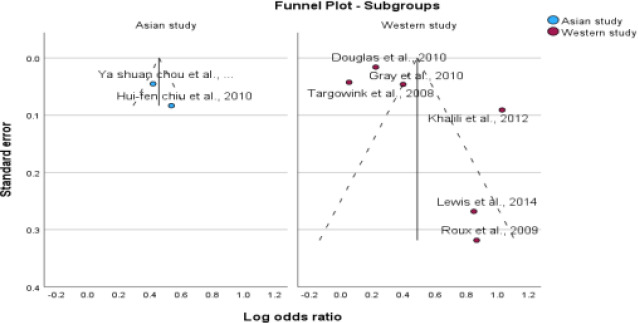
Subgroup analysis based on ethnicity

**Figure 8:**
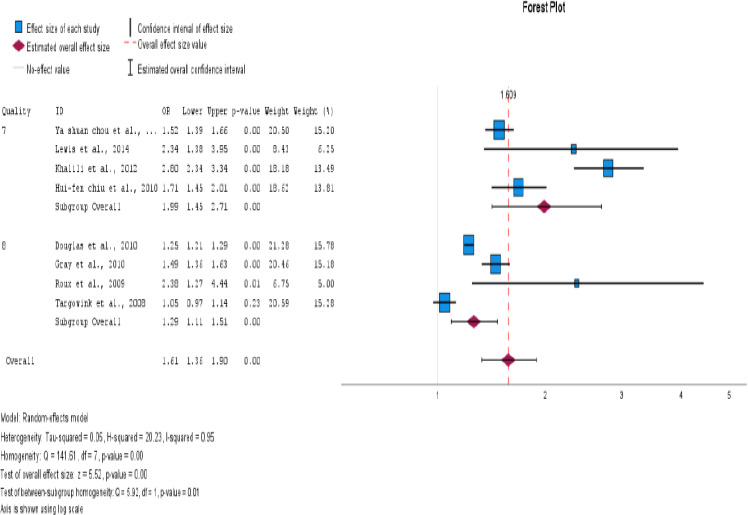
Subgroup analysis based on quality

**Figure 9:**
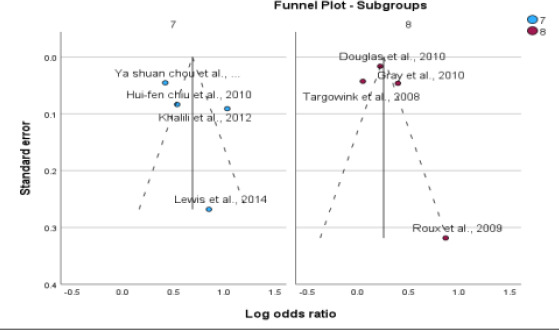
Subgroup analysis based on quality

**Figure 10:**
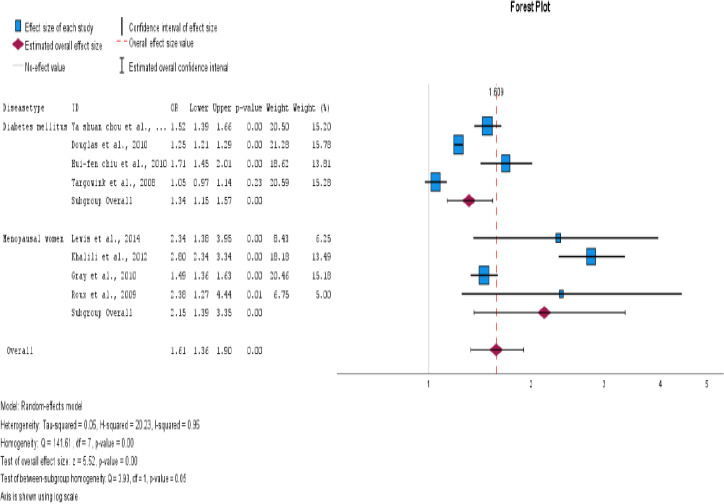
Subgroup analysis based on disease type (type 2 DM and postmenopausal women)

**Figure 11:**
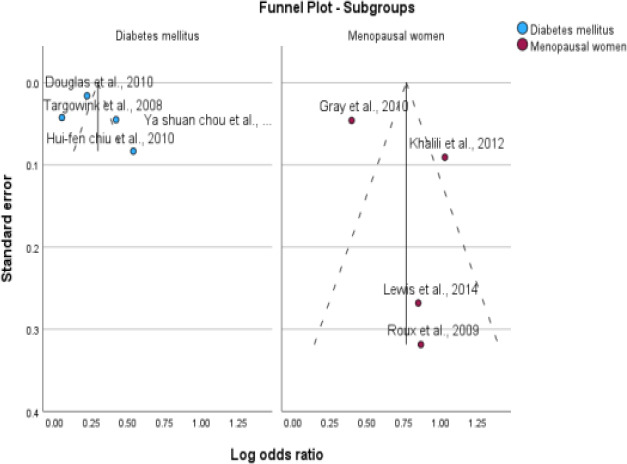
Subgroup analysis based on disease type (type 2 DM and postmenopausal women)

**Figure 12:**
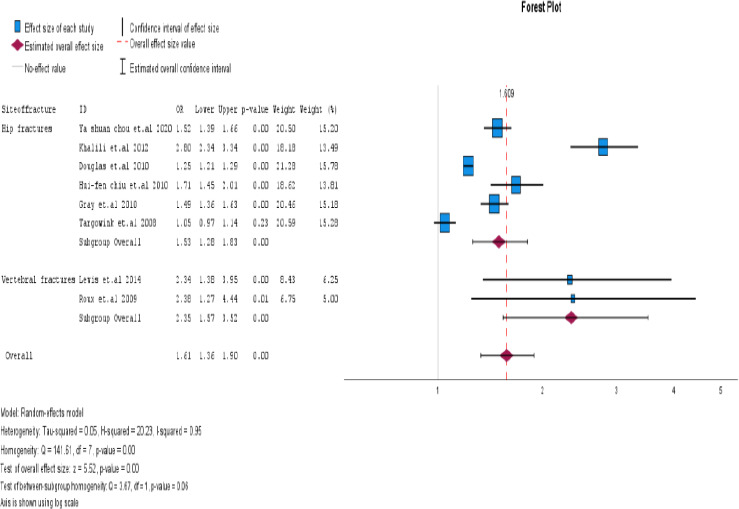
Subgroup analysis based on fracture site (Hip & Vertebral)

**Figure 13:**
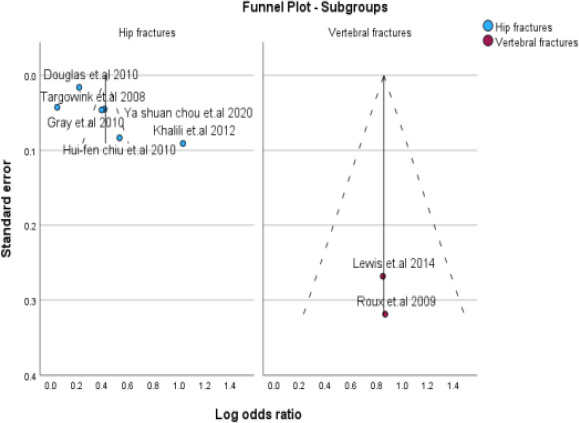
Subgroup analysis based on fracture site (Hip & Vertebral)

We conducted subgroup analyses to investigate the impact of PPI use on the risk of fractures in postmenopausal women and individuals with type 2 diabetes. The study design, ethnicity, fracture site, and methodological quality were examined to identify potential sources of heterogeneity. The aim of this study was to gain a more comprehensive understanding of the impact of these factors on fracture risk. For subgroup analysis, the information presented in Table 4, all forest plots, and funnel plots are displayed in Figures 4–13.

**Table 4:** Overall effect estimates for PPI use in type 2 DM and menopausal women according to study characteristics/subgroup analysis

Study	No. of studies	Random-effect model: OR (95%CI)	Heterogeneity between studies	
			P-Value	I^2^ -Value
**All**	8	1.60(1.35-1.90)	<.001	95%
**Subgroup analysis**				
**Study design**				
**Cohort**	6	1.73(1.32-2.26)	<.001	95.6%
**Case-control**	2	1.44(1.06-1.96)	0.019	92.7%
**Ethnicity**				
**Asian**	2	1.57(1.41-1.75)	<.001	34.5%
**western**	6	1.62(1.30-2.02)	<.001	95.7%
**Quality**				
**High (>7)**	5	1.29(1.10-1.50)	<.001	91.3%
**Low (7)**	3	1.98(1.43-2.70)	<.001	92%
**Disease type**				
**Menopausal women**	4	2.15(1.38-3.34)	<.001	92.7%
**Type 2 DM**	4	1.34(1.14-1.57)	<.001	93.8%
**Site of fractures**				
**Hip fracture**	6	1.53(1.28-1.82)	<.001	96.2%
**Vertebral fracture**	2	2.35(1.57-3.51)	0.966	0%

The impact of PPI use in type 2 DM and postmenopausal women with fracture risk was examined. It has been found that postmenopausal women who use PPIs are at a higher risk of developing fractures. The OR was 2.15, with a 95% CI of 1.38-3.34 and an I^2^ value of 92%. This indicates a statistically significant association between the two variables, with a p<0.001.

Studies have shown that PPIs are associated with an increased risk of fractures, particularly in the hip and vertebral regions. The OR for hip fracture was 1.53, with a 95% CI of 1.28-1.83. The I2 statistic was 96%, and the p<0.001. The OR for vertebral fracture was 2.35 with a 95% CI of 1.57-1.90. The I2 statistic was 0%, and the p = 0.966. This finding suggests that PPI use is more likely to cause hip fractures than vertebral fractures.

In addition, individuals with type 2 diabetes have an increased odds ratio of developing fractures, with a value of 1.34 and a 95% CI of 1.14-1.57. The I^2^ value for this association was 93%, suggesting a statistically significant association. The p value for this relationship was < 0.001. For postmenopausal women, the OR was 2.15 with a 95% CI (1.36-1.90), I2=92%, and p<0.001. When both factors were present, the risk of fracture development was approximately 74.2%. However, it is essential to consider that other factors such as obesity, diet, and comorbidities could also contribute to an increased risk of fractures.

Six cohort studies and two case-control studies were conducted to evaluate the risk of fractures associated with PPI use in patients with type 2 DM and postmenopausal women. The results revealed a significant association between PPI use and fracture risk. The odds ratio for cohort studies was OR 1.73, 95% CI (1.32-2.26), I2=95%, and p<0.001. For case-control studies, the was OR 1.44 with 95% CI (1.06-1.96), I^2^=92%, and a p = 0.019. Cohort studies have demonstrated a significant association between PPI use and fracture risk.

The studies included in the analysis were conducted in Asian and Western countries. Of the six studies conducted in Western countries, an association was found between PPI therapy and fracture risk, with a pooled OR of 1.62, 95% CI (1.41-1.75), I^2^=95%, and p<0.001. However, in Asian countries, no such association was observed, with a pooled odds value of 1.57, CI (1.41-1.75), I^2^=34%, and p<0.001.

Grouping of studies by methodological quality showed a significantly increased fracture risk with PPI use in both high-quality studies (OR 1.29, 95% CI (1.10-1.50), I2 = 91 %, and p<0.001) and low-quality studies (OR 1.98, 95% CI (1.45-2.70), I2 = 92%, and p = 0.001). Based on study quality, the number of variables was adjusted for (>7 vs. 7). Table 4 provides a detailed description of the subgroup analysis.

### Publication Bias

No significant asymmetry was found in the funnel plots, and the results of the Egger's test were not significant. The P value for bias in the Egger's test was 0.45 for PPI use, indicating no significant influence of publication bias among the included studies.

## Discussion

This meta-analysis investigated the association between proton pump inhibitors (PPIs) use and fracture risk in type 2 diabetic mellitus and postmenopausal women, analyzing six cohort studies and two case-control studies, with diverse methodologies and sample populations. In a meta-analysis of all studies, there was significantly associated between PPI use and an elevated risk of fracture. Thus, the pooled effect estimate was OR=1.61 with a 95% CI of 1.35-1.90 and a p<0.001. As a result, this observational study demonstrated that the use of PPIs in Type 2 diabetes and menopausal women is associated with an increased risk of fractures. Individuals taking proton pump inhibitors (PPIs) have a higher risk of fracture than those who do not. The combined odds ratio from both case-control and cohort study designs indicated a significant association between PPI use and increased fracture risk among menopausal women and patients with type 2 diabetes.

Further analysis of various subgroups revealed a high risk of fracture. The risk was even higher when both conditions were present, such as in menopausal women with type 2 diabetes, where the estimated risk of fractures was significantly increased, suggesting a potential additive effect. Interestingly, studies conducted in Western countries showed a more significant association between PPI use and fracture risk than those conducted in Asian countries. Both high- and low-quality studies have found an increased risk of fractures associated with PPI use. The fracture site showed a significant increase in the risk of fractures, mainly in the hip, compared with the vertebrae, which reinforces the consistency of this association across diverse methodological approaches. This finding warrants further investigation into the potential variations.

In a combined analysis of all trials, there was a substantial relationship between PPI use and increased risk of fractures. Although the precise impact of acid inhibition on bone mineral density over time is uncharacterized, there are several physiologic mechanisms by which using proton pump inhibitors could affect bone mineral metabolism^15^. Calcium absorption is highly variable and depends on several factors, including vitamin D status, total daily intake, dose, and concurrent food intake. This direct effect on the bone could counteract the potential negative impact of calcium malabsorption^16^. During menopause, the levels of blood estrogen can decrease by up to 90%. This decrease may result in greater bone resorption than production. As a result, rapid bone loss and calcium outflow from the skeleton to the extracellular fluid occur. The rate of bone turnover increases as estrogen levels decrease during menopause, which is the main reason for increased bone loss in total body calcium.

Ultimately, this leads to bone loss. Bone loss causes the skeletal microarchitecture to deteriorate, thereby increasing the risk of fracture. Apart from menopause and osteoporosis, other conditions may require alterations in bone metabolism^17^. However, poor calcium metabolism is associated with type 2 diabetes, whih can exacerbate bone fragility. PPI-induced Hip fracture (HFx) and type 2 DM should be examined to understand the etiology and link between the two, as parathyroid hormone (PTH) elevation is associated with glucose metabolism problems. According to Ya-Shuan et al.,^18^ the impact of PPI use in patients with type 2 DM and fracture risk was assessed in this study. Older age, female sex, and a higher Charlson comorbidity index (CCI) score are predictive factors for HFx and may indicate a more precarious medical condition and an increased risk of osteoporosis. PPI use was a significant and independent predictor of HFx in patients with type 2 DM, with an adjusted HR of 1.41. Nevertheless, no correlation was found between PPI dose and HFx risk. Prior studies have linked PPI therapy to an increased fracture risk.

Targowink et al. reported that the use of PPIs for five or more years continuously increases the risk of hip and osteoporotic fractures. However, patients who use them for less than six years are not at a significant risk of osteoporosis-related fractures compared to those who do not use them. Similarly, they are not at an increased risk of hip fracture if they use them for four or fewer years. A recent study by Yang et al. found that patients who had experienced hip fractures were 1.21 times more likely to have used a PPI for over a year and 1.59 times more likely to have used them for four years or more. These results suggest a possible link between long-term PPI use and an increased risk of hip fractures. In another study by Roux et al.,^19^ there is evidence to indicate that the use of omeprazole in postmenopausal women increases the risk of vertebral fracture, regardless of other risk factors such as age, low bone density, and previous fractures. The link between omeprazole use and the occurrence of vertebral fractures was significant and comparable to the risk associated with previous fractures. This is consistent with findings from case-control studies, which have shown that using PPIs for over a year increases the risk of hip fracture.

In their research on drug usage, Roux et al. studied omeprazole, while Shelly L. Gray et al. focused on esomeprazole magnesium, lansoprazole, omeprazole magnesium, pantoprazole sodium, and rabeprazole^16^,^19^. The other studies did not specify these drugs individually and referred to them collectively as PPI. None of the eight studies detailed the dosages used. Additionally, the duration of PPI use varied across studies. The follow-up periods for patients on PPI were as follows: Roux et al.^19^ had a duration of 6.1 years, Shelly L. Gray et al.^16^ had 7.8 years, Hameed Khalili et al.^1^ had 9 years, Ya-shuan Chou et al.^18^ had 5 years, Lewis et al.^27^ had 5 years, Douglas et al.^28^ had more than 2 years, Hui-Fen Chiu et al.^2^ had 2 years, and Targownik et al.^15^ had 7 years. However, because each study was conducted in different countries, there were variations in regional healthcare practices, resulting in greater heterogeneity in the current study.

After conducting a subgroup analysis, we found that study design, ethnicity, methodological quality, and fracture site did not significantly impact the results. The estimates were consistent across studies with different ethnicities, including Asian and Western countries. However, there was a notable variation in the type of disease and fracture site. Multiple subgroup analyses revealed that the results were robust.

Our study had some limitations that deserve further discussion. First, this meta-analysis used data from epidemiological studies (case-control and cohort studies). The observational design limits causal inference, and potential confounding variables must be considered. Second, it establishes an association, not necessarily causation. Other factors, such as obesity, diet, and other comorbidities, may play a role. Third, the included studies were conducted only in Asian and Western countries and no evidence was found on different continents. Fourth, we only searched for existing publications concerning PPI usage in disease type (type 2 DM and menopausal women) and fractures, which would lead to the inclusion of mere epidemiological studies of drug safety. This study included case-control and cohort studies to investigate the relationship between PPIs and fractures. However, exploring and reporting original randomized clinical trials regarding PPI efficacy and adverse effects are essential.

Further studies are required to determine the clinical importance of our findings, as this meta-analysis has limitations. These findings suggest that PPIs should be prescribed with caution in type 2 diabetic and menopausal women, especially those with additional fracture risk factors. It is essential to consider individual risk-benefit profiles, alternative treatment options, and potential risk factors for fractures before prescribing PPIs to this demographic. Patients undergoing long-term PPI therapy, particularly those with identified risk factors, should be monitored for possible fractures. These findings have significant clinical implications and underline the need for careful and individualized assessments before prescribing PPIs to this group of patients. Other limitations include the need for more prospective studies to determine the effects of PPI use on fracture risk. Nonetheless, our findings show patients and physicians that PPI use negatively affects bone mineral health in disease conditions. In our study, we analyzed eight articles that met our research requirements. We pooled the data from these articles and used the OR to obtain the results. It is important to note that this study alone cannot provide conclusive results. However, our analysis provides valuable information on how these medications affect individuals over an extended period of time in disease conditions. The results of our study are significant and add to existing literature on the safety of PPI use. These insights are essential for clinical practice. Although a research protocol was developed prior to this meta-analysis, it was not registered in advance with PROSPERO or any other public databases. This could be considered a limitation, as prospective registration helps reduce bias and improves methodological transparency. Nevertheless, we strictly followed PRISMA guidelines during the review process to maintain scientific rigor.

## Conclusion

Our meta-analysis was conducted to investigate the impact of PPI use and fracture risk in patients with disease conditions such as menopausal women and type 2 DM. Research findings have indicated that extended use of PPIs in patients with specific medical conditions can elevate the risk of fractures in these patient populations. Therefore, we strongly recommend that long-term users with medical conditions seek appropriate medical consultation before continuing PPI therapy. It is crucial to point out that PPIs are often prescribed without adequate assessment of their necessity. Therefore, it is essential to evaluate the individual requirements of each patient for PPI usage. Healthcare providers should strive to reduce PPI use in clinical settings where PPI therapy is ongoing.
